# Association between aortic peak wall stress and rupture index with abdominal aortic aneurysm–related events

**DOI:** 10.1007/s00330-023-09488-1

**Published:** 2023-03-10

**Authors:** Tejas P. Singh, Joseph V. Moxon, T. Christian Gasser, Jason Jenkins, Michael Bourke, Benard Bourke, Jonathan Golledge

**Affiliations:** 1 Queensland Research Centre for Peripheral Vascular Disease, College of Medicine and Dentistry, James Cook University, Townsville, Queensland 4811 Australia; 2 The Department of Vascular and Endovascular Surgery, The Townsville University Hospital, Townsville, Queensland Australia; 3 The Australian Institute of Tropical Health and Medicine, James Cook University, Townsville, Queensland Australia; 4 Department of Engineering Mechanics, KTH Solid Mechanics, KTH Royal Institute of Technology, Stockholm, Sweden; 5 Department of Vascular and Endovascular Surgery, Royal Brisbane and Women’s Hospital Brisbane, Herston, Queensland Australia; 6 Gosford Vascular Services Gosford New South Wales Australia, Gosford, Australia; 7 The School of Biomedical Sciences & Pharmacy, The University of Newcastle, Newcastle, New South Wales Australia

**Keywords:** Peripheral vascular diseases, Vascular diseases, Aortic aneurysm, abdominal

## Abstract

**Objective:**

The aim of this study was to assess whether aortic peak wall stress (PWS) and peak wall rupture index (PWRI) were associated with the risk of abdominal aortic aneurysm (AAA) rupture or repair (defined as AAA events) among participants with small AAAs.

**Methods:**

PWS and PWRI were estimated from computed tomography angiography (CTA) scans of 210 participants with small AAAs (≥ 30 and  ≤ 50 mm) prospectively recruited between 2002 and 2016 from two existing databases. Participants were followed for a median of 2.0 (inter-quartile range 1.9, 2.8) years to record the incidence of AAA events. The associations between PWS and PWRI with AAA events were assessed using Cox proportional hazard analyses. The ability of PWS and PWRI to reclassify the risk of AAA events compared to the initial AAA diameter was examined using net reclassification index (NRI) and classification and regression tree (CART) analysis.

**Results:**

After adjusting for other risk factors, one standard deviation increase in PWS (hazard ratio, HR, 1.56, 95% confidence intervals, CI 1.19, 2.06; *p* = 0.001) and PWRI (HR 1.74, 95% CI 1.29, 2.34; *p* < 0.001) were associated with significantly higher risks of AAA events. In the CART analysis, PWRI was identified as the best single predictor of AAA events at a cut-off value of  > 0.562. PWRI, but not PWS, significantly improved the classification of risk of AAA events compared to the initial AAA diameter alone.

**Conclusion:**

PWS and PWRI predicted the risk of AAA events but only PWRI significantly improved the risk stratification compared to aortic diameter alone.

**Key Points:**

*• Aortic diameter is an imperfect measure of abdominal aortic aneurysm (AAA) rupture risk.*

*• This observational study of 210 participants found that peak wall stress (PWS) and peak wall rupture index (PWRI) predicted the risk of aortic rupture or AAA repair.*

*• PWRI, but not PWS, significantly improved the risk stratification for AAA events compared to aortic diameter alone.*

**Supplementary Information:**

The online version contains supplementary material available at 10.1007/s00330-023-09488-1.

## Introduction

Abdominal aortic aneurysm (AAA) rupture is responsible for approximately 200,000 deaths per year worldwide [1–3]. Maximum AAA diameter is the most established method of estimating AAA rupture risk and is used by clinicians to help select patients for elective AAA repair [1]. Current guidelines recommend that small (< 55 mm diameter in men and  < 50 mm in women) asymptomatic AAAs are managed conservatively while larger AAAs are considered for surgical repair [4]. Some large AAAs do not rupture during a patient’s lifetime [5], while 1–2% of small AAAs rupture per year [6], suggesting that AAA diameter is not a perfect measure of rupture risk. Other methods of estimating AAA rupture risk have been proposed including AAA volume, functional imaging (e.g., positron emission tomography), and circulating biomarkers; however, there has been limited uptake of these methods in clinical practice [7, 8]. More accurate and user-friendly methods to estimate AAA rupture risk could benefit patient management.

**Fig. 1:**
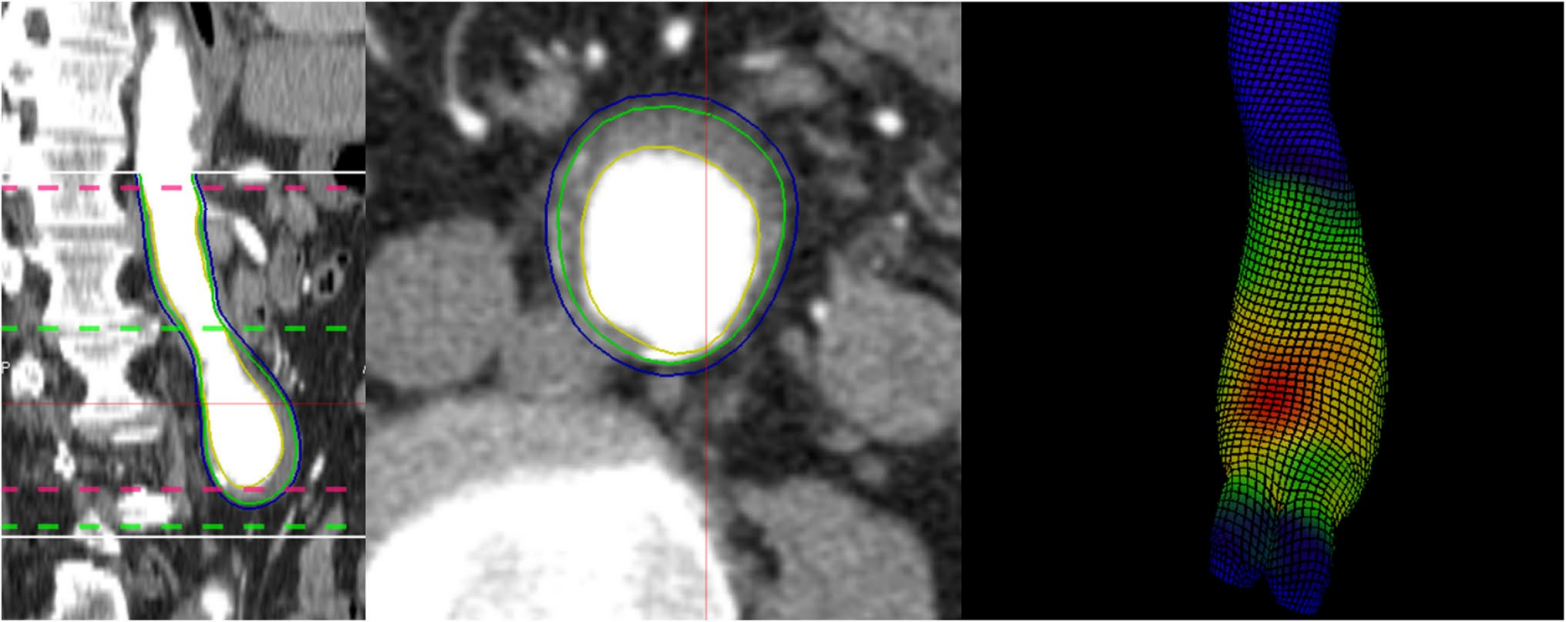
Example of a three-dimensional (3D) segmentation produced using finite element analysis on the computed tomography image of an AAA

Finite element analysis (FEA) is an established engineering technique that can non-invasively estimate the peak tensile stress within the AAA wall (peak wall stress) from computed tomography images (Fig. 1) [9]. A recent prospective study reported that the dimensionless ratio of wall stress and wall strength (defined as the ABR) significantly predicted the risk of AAA rupture or repair (AAA events) independent of other risk factors [10]. The ABR was computed by FEA of three-dimensional CT images of the aorta and uniquely incorporated patient-specific aortic wall thickness estimates [11, 12]. Aortic wall thickness measurement required magnetic resonance imaging (MRI) which is not routinely performed in AAA management [10, 13]. Furthermore, the method employed in that study has not been widely studied [10, 13], and required multiple software packages to perform the biomechanical analysis [13].

A prior meta-analysis [9] found that the peak wall rupture index (PWRI, i.e., the ratio between aortic wall stress and strength estimated assuming a constant wall-thickness) [14, 15], but not peak wall stress (PWS), was significantly greater in ruptured than asymptomatic intact AAAs of similar diameter. Both measurements can be estimated from contrast-enhanced computed tomography angiograms (CTA) using one software package [15, 16] with good repeatability [9, 11, 17, 18]. The studies included in prior reviews [9, 19] were of cross-sectional design and ostensibly included patients with large AAAs. There is a lack of observational studies investigating the association between PWS and PWRI with the future risk of AAA events among individuals with small AAAs (maximum orthogonal aortic diameter of  ≥ 30 and  ≤ 50 mm) [10, 20]. Such data is required to assess whether PWS and PWRI can predict the risk of future AAA events amongst people with a low risk of AAA rupture [10, 20]. Furthermore, it is unclear whether PWS and PWRI can improve the classification of risk of AAA events in comparison to using AAA diameter alone. The primary objective of this prospective observational study was to assess whether baseline PWS and PWRI were independently associated with the risk of future AAA events among individuals with small AAAs. The secondary objective was to examine whether PWS and PWRI significantly improved the stratification of risk of AAA events over using AAA diameter alone.

## Methods

### Study design and participants

Participants were recruited from sites across Australia, the USA, and Netherlands between 29/05/2002 and 24/06/2016 via two sources. Firstly participants were included from those taking part in an ongoing multi-centre prospective cohort study of people with a range of peripheral vascular diseases [20, 21]. Secondly, participants were included from an international multi-centre trial of patients with small AAAs, which showed that telmisartan did not slow AAA growth, as previously reported [22]. Participants were eligible for the current study if they had a small infra-renal (maximum orthogonal aortic diameter of  ≥ 30 and  ≤ 50 mm) asymptomatic intact AAA which had been imaged by a CTA [11]. CTAs needed to have a slice thickness of 3 mm or less and visualise the whole infra-renal aorta including the bifurcation into the common iliac arteries [11]. Patients with symptomatic or ruptured AAAs were excluded. Written informed consent was obtained from all participants. The study was performed in accordance with the Helsinki declaration and ethical approval was granted from institutional ethics committees (HREC/09/QTHS/117; HREC/14/QTHS/203; HREC/13/QTHS/125) [21–23].

### Participant characteristics

Risk factors and medication prescription records were collected at the time of enrolment into the study [21–23]. Coronary heart disease (CHD) was defined by a history of myocardial infarction, angina, or coronary revascularisation [24, 25]. Current smoking was defined as smoking within the last month based on participants’ history. Hypertension, diabetes, and chronic obstructive pulmonary disease (COPD) were defined by a prior diagnosis or treatment for these conditions [21–23]. Blood pressure was measured at recruitment using a digital monitor (Omron Intellisense, HEM-907) after participants had rested supine for a 20-min period [23, 26]. Prescriptions for aspirin, anticoagulants, statins, calcium channel blockers, beta-blockers, and metformin were obtained from medical records.

#### CTA

CTAs were performed using institutional scanners at each participating hospital with departmental-specific image acquisition protocols as previously reported [11, 21–23]. All CTAs were transferred to the core imaging reading site (Townsville, Australia), where they were analysed using the Philips MxView Visualisation Workstation using the Advance Vessel Analysis application (v7) [11, 21–23]. This programme was used to estimate maximum orthogonal aortic diameter using a validated protocol as previously described [8, 11, 27]. A region of interest (ROI) was selected, which included the region marked by the slice inferior to the origin of the lowest renal artery (excluding accessory arteries) to the slice superior to the aortic bifurcation. Within this ROI, the aorta was scouted by the operator to identify the region of maximal diameter by performing many measurements [8, 11, 27]. Anterior–posterior outer-to-outer orthogonal diameters were estimated by tracing the lumen of the infrarenal aorta and measuring perpendicular to this axis. The measurement was recorded to the nearest 0.1 mm [8, 11, 27]. The reproducibility of this method has been previously assessed (coefficient of variation  < 4%) [27].

### Biomechanical analysis

PWS and PWRI were estimated from the FEA of CTAs using commercially available software (A4 Research 5.0, VASCOPS GmbH) as previously described [9, 11, 14]. PWS estimated the maximum tensile stress to which the aortic wall was subjected based on AAA morphology and blood pressure (BP). PWRI estimated the maximum ratio between wall stress and the estimated local aortic wall strength [9, 11]. Three-dimensional (3D) reconstructions of the AAA were created from an ROI using the boundaries as defined earlier. The 3D model was processed into a hexahedral mesh to prevent volume locking of incompressible solids [11, 14]. AAA wall strength was estimated using a statistical model incorporating intra-luminal thrombus thickness, AAA diameter, and sex as previously described [11, 14, 16]. Wall strength values related to the variables included in this model were estimated from tensile testing of human AAA wall specimens, as described previously [16, 28]. The AAA FEA model was pressurised by inputting BP, which in turn estimated the mechanical stress on the aortic wall [9, 11, 14, 16]. The main analysis used patient-specific BP at recruitment to compute PWS and PWRI. A sensitivity analysis was performed using a standardized BP of 140/80 mmHg consistent with the approach of prior studies [9, 11]. Biomechanical analyses were performed by a medical doctor who received 12 months of training in FEA. The intra-observer reproducibility of PWS in asymptomatic intact AAAs has previously been reported (coefficient of variation 2.7%) [11].

### Definition and assessment of outcome

The primary outcome was AAA events defined as AAA rupture or repair [29, 30]. This was recorded through prospective follow-up which included clinical reviews, medical record reviews, and linked data on inpatient admissions as previously described [21–23]. Decisions regarding the requirement for surgical repair were at the discretion of the treating vascular surgeon but were consistent with current international guidelines [1, 4]. Surgical repair was performed by a vascular surgeon. Participants were censored at the first outcome event, or at the date of the last review or linked data request if an outcome event did not occur.

### Sample size

The sample size for the present study was based on the planned Cox regression analyses to assess the associations between PWS/ PWRI and the risk of AAA events. Based on previous studies of patients with small AAAs, the rate of AAA events was estimated to be 20% over 2 years [10, 23, 31]. The Cox proportion hazard analyses were planned to include 3 covariates (AAA diameter, statin prescription, and age). It was estimated that at least 200 individuals would lead to a well-powered analysis in order to attain at least 10 outcome events per degree of freedom according to Monte-Carlo simulations [32].

### Data analysis

Nominal data were compared between groups using the Pearson *χ*^2^ test. Most continuous variables were not normally distributed according to Q–Q plots and Kolmogorov–Smirnov testing and therefore non-parametric Mann–Whitney U tests were used to compare groups. Kaplan–Meier curves with the log-rank test were used to compare the proportion of participants having AAA events. Cox proportional hazard analyses were undertaken to assess the association between PWS and PWRI with AAA events. To examine whether PWS and PWRI were independently associated with AAA events Cox proportional hazard analyses were adjusted for age, male sex, statin prescription, and AAA diameter. These variables were selected for adjustment as they were different (*p* < 0.100) between participants who had an AAA event and those who did not. Results were presented as hazard ratios (HR) and 95% confidence intervals (CI). HRs were expressed per 1 standard deviation increase in PWS or PWRI. A sub-analysis was performed which was restricted to female participants. A correlation matrix of coefficients in the Cox models was used to assess if there was co-linearity between variables included in the regression analyses [33, 34]. A correlation coefficient  ≥ 0.60 was considered to indicate a high likelihood of co-linearity and was not found with any of the variables included in the final models [33–35]. Whether PWS and PWRI with or without clinical risk factors significantly improved stratification of risk of AAA events over using AAA diameter alone was examined using the net reclassification index (NRI) [22]. Clinical risk factors included were diabetes and current smoking as these are recognised risk factors for AAA growth [4, 36]. Classification and regression tree analysis (CART) was used to determine the optimal predictive cut-off of variables that were found to best stratify the risk of AAA events. The sample was segregated according to a decision tree consisting of progressive binary splits as previously described [37]. Every value of each predictive variable was considered as a potential split and the optimal split was based on the impurity criterion [38]. The maximum *p* value for a split was set at 0.050. A sensitivity analysis was performed in which a standardized BP of 140/80 mmHg was used to calculate PWS and PWRI. Data were analysed using the Stata v16.1 (StataCorp LP) software package. *p* values of  < 0.05 were accepted to be significant for all analyses.

## Results

### Participant characteristics

A total of 210 participants were included and followed up for a median of 2.0 (interquartile range [IQR] 1.9, 2.8) years. During this time, 45 (21%) participants had an AAA event including 43 who had an AAA repair and 2 that had an AAA rupture. Repairs included 36 endovascular and 7 open surgical repairs. The baseline characteristics of participants in relation to whether they later had an AAA event are presented in Table 1. Participants who had an AAA event had a significantly larger initial maximum orthogonal aortic diameter (median [IQR], 44.4 [40.8, 47.0] vs 40.2 [36.5, 42.8] mm;* p* < 0.001) and were significantly younger at the time of recruitment than those not having an event (*p* = 0.038). No significant differences in sex, current smoking, diabetes, CHD, BP, and other risk factors between groups were identified (see Table 1).

**Table 1:** Baseline characteristics of participants with small AAAs who experienced an AAA event and those who did not

	No AAA event (*n* = 165)	AAA event (*n* = 45)	*p* value
Age	74 (68, 80)	71 (67, 77)	0.038
Male sex	150 (91)	37 (82)	0.098
Current smoking	39 (24)	13 (29)	0.469
Ever smoker	155 (94)	43 (96)	0.679
Hypertension	86 (52)	24 (53)	0.885
Diabetes	33 (20)	7 (16)	0.501
CHD	67 (41)	18 (40)	0.941
COPD	46 (28)	10 (22)	0.447
Aspirin	93 (56)	29 (64)	0.330
Anticoagulation	17 (10)	3 (7)	0.461
Statin	104 (63)	34 (77)	0.084
Calcium channel blocker	28 (17)	5 (11)	0.338
Beta blocker	43 (26)	11 (24)	0.826
Metformin	21 (13)	5 (11)	0.770
Systolic blood pressure (mmHg)	137 (125, 150)	135 (125, 146)	0.423
Diastolic blood pressure (mmHg)	78 (71, 85)	76 (70, 83)	0.413
Maximum orthogonal diameter (mm)	40.2 (36.5, 42.8)	44.4 (40.8, 47.0)	< 0.001
PWS (kPa)	157.4 (142.9, 180.1)	182.5 (153.6, 209.4)	< 0.001
PWRI	0.352 (0.308, 0.404)	0.415 (0.363, 0.514)	< 0.001

*AAA*, abdominal aortic aneurysm; *PWS*, peak wall stress; *kPa*, Kilopascals; *PWRI*, peak wall rupture index; *CHD*, coronary heart disease; *COPD*, Chronic obstructive pulmonary disease. Continuous data are presented as median [interquartile range] and were compared using the Mann–Whitney U test. Nominal data are presented as numbers (%) and were compared using Pearson’s *χ*^2^ test. *p* values highlighted in boldface indicate significant differences

### Association between PWS and PWRI at entry and AAA events

**Fig. 2:**
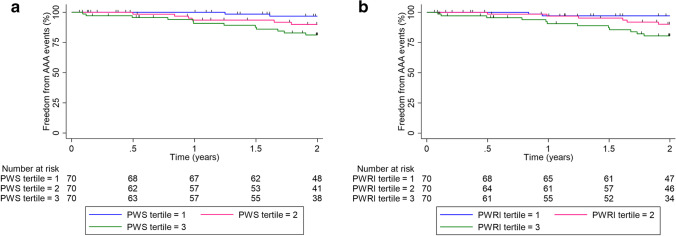
Kaplan–Meier curves illustrating the freedom from AAA events according to tertiles of peak wall stress (PWS) and peak wall rupture index (PWRI). **a** PWS; **b** PWRI. Differences between both groups compared using the log-rank test (*p* = 0.001 and *p* < 0.001 for PWS and PWRI respectively)

PWS and PWRI at entry were significantly greater in participants who later had an AAA event compared to those that did not (*p* < 0.001 and *p* < 0.001 respectively; see Table 1). Figure 2 illustrates the proportion of participants who had an AAA event in relation to the tertiles of PWS and PWRI measured at entry. A greater proportion of participants grouped in tertile 3 of PWS and PWRI had an AAA event compared to individuals in tertile 1 (log-rank test *p* < 0.001 and *p* < 0.001 for PWS and PWRI respectively). Findings from the Cox proportional hazard analysis are reported in Table 2. In the unadjusted analysis, both higher PWS and PWRI at entry were associated with a significantly higher risk of an AAA event. In the adjusted analysis both higher PWS (HR 1.56, 95% CI 1.19, 2.06; *p* = 0.001) and PWRI (HR 1.74, 95% CI 1.29, 2.34; *p* < 0.001) were associated with a significantly increased risk of AAA events. In the sub-analysis restricted to female participants, high PWRI, but not PWS, was associated with an increased risk of AAA events (Supplementary Table 1).

**Table 2:** Association between PWS and PWRI with AAA events in individuals with small AAA

	AAA events (AAA rupture or repair)		
	Hazard ratio (HR) †	95% CI	*p* value
	Unadjusted analysis		
PWS (kPa)	1.89	1.52, 2.33	< 0.001
PWRI	1.92	1.58, 2.34	< 0.001
	Adjusted analysis*		
PWS (kPa)	1.56	1.19, 2.06	0.001
PWRI	1.74	1.29, 2.34	< 0.001

*AAA*, abdominal aortic aneurysm; *PWS*, peak wall stress; *kPa*, Kilopascal; *PWRI*, peak wall rupture index. *Adjusted for variables that were found to be different (*p* < 0.100) between participants who had events and those who did not have events (i.e., AAA diameter, statin prescription, age, and male sex); † Hazard ratios expressed per 1 standard deviation increase in PWS or PWRI

### Ability of PWS and PWRI to improve stratification of risk of AAA events

**Fig. 3:**
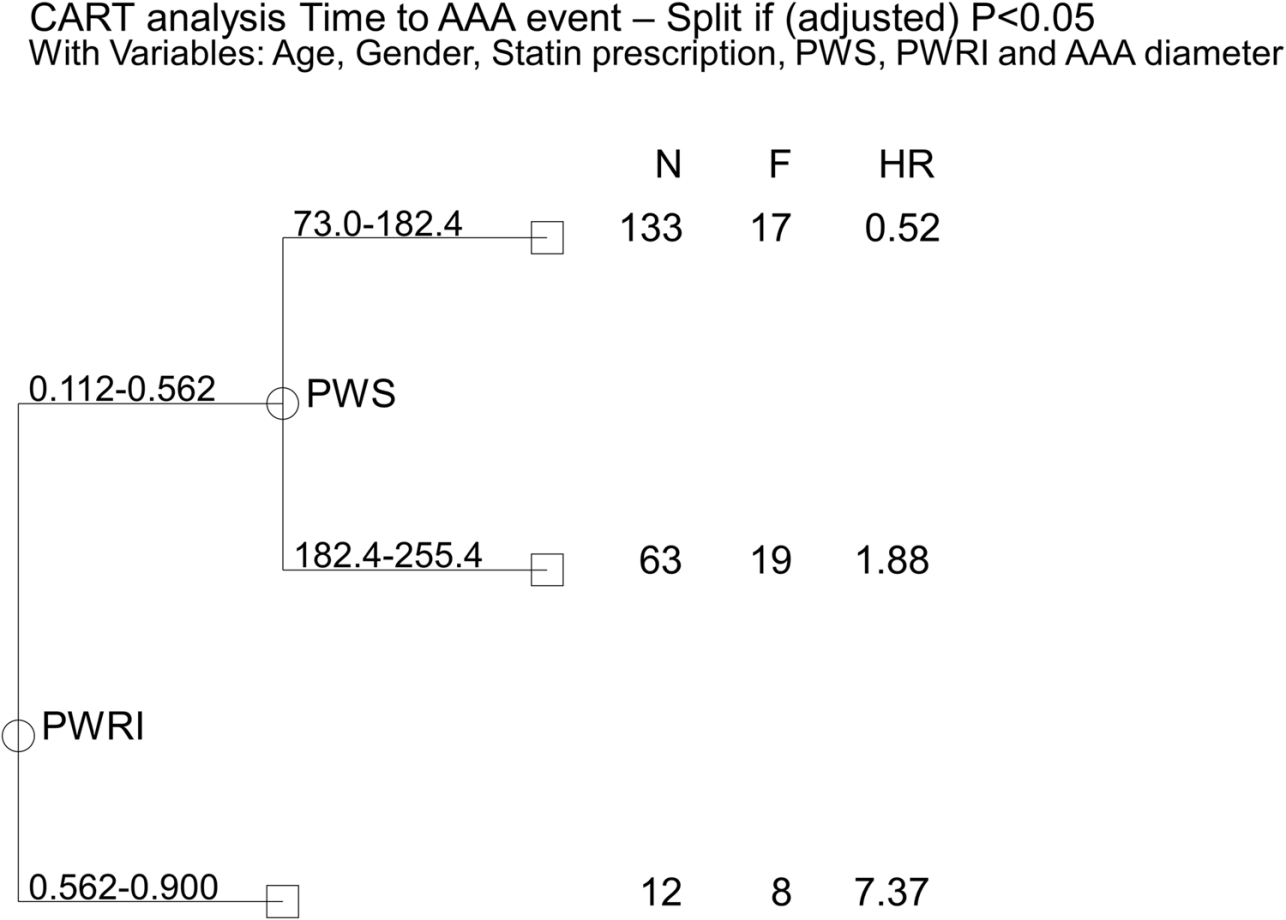
Classification and regression tree analysis (CART) for AAA events. Variables different (*p* < 0.100) between participants who had events and those who did not have events (age, statin prescription, peak wall stress [PWS], peak wall rupture index [PWRI], and AAA diameter) were entered into the analysis. The maximum *p*-value for a split was set at 0.050. N, numbers of individuals in subgroup; F, events; HR, hazard ratio

**Fig. 4:**
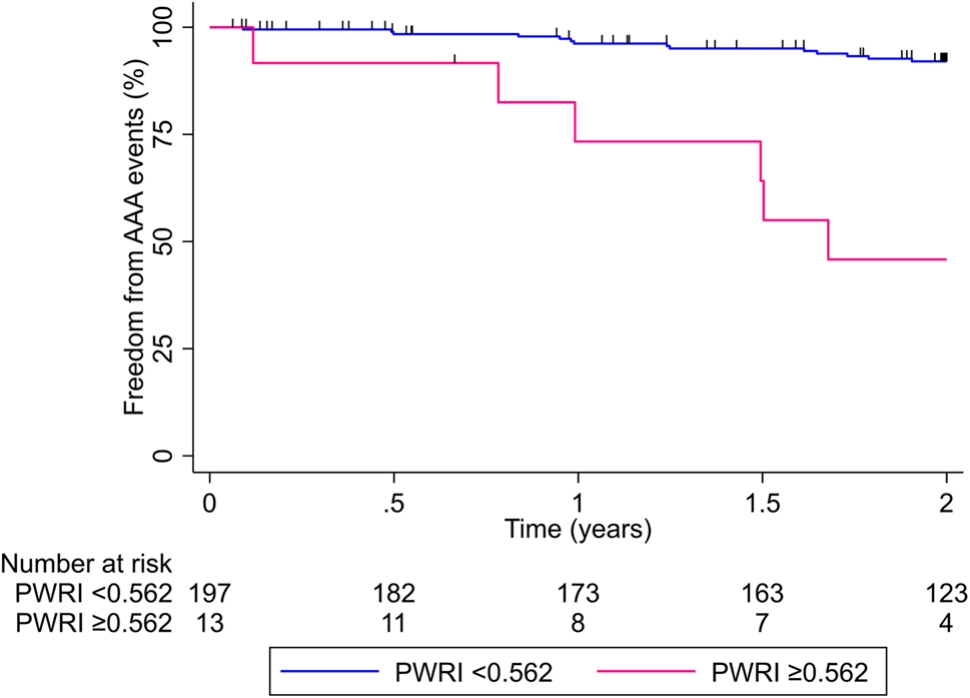
Freedom from AAA events stratified by initial peak wall rupture index (PWRI). The optimal PWRI cut-off was determined by classification and regression tree analysis. Differences between both groups were compared using the log-rank test (*p* < 0.001)

PWRI (NRI 0.42 95% CI 0.09, 0.75; *p* = 0.013), but not PWS (NRI 0.26 95% CI − 0.07, 0.59; *p* = 0.124), significantly improved the classification of risk of AAA events compared to AAA diameter alone. Models incorporating clinical risk factors, AAA diameter, and PWRI (but not PWS) significantly improved the classification of risk of AAA events compared to AAA diameter alone (Table 3). All baseline variables that were different between participants that did and did not have an AAA event (*p* < 0.100) were entered into the CART analyses. PWS and PWRI contributed to the stratification of risk of AAA events, estimated between HR 0.52 and 7.37. PWRI was identified as the best single risk stratification measure for AAA events, using a cut-off value of 0.562 (Fig. 3). Participants with PWRI  ≥ 0.562 were significantly more likely to experience a AAA event than those with PWRI  < 0.562 (Cox proportional HR for AAA events: 5.55; 95% CI 2.67, 11.57, *p* < 0.001; Fig. 4).

**Table 3:** Discrimination and reclassification using PWS and PWRI for AAA events

Models	NRI (95% CI)	*p* value
AAA diameter (reference)	-	-
AAA diameter + PWS	0.26 (−0.07, 0.59)	0.124
AAA diameter + PWRI	0.42 (0.09, 0.75)	0.013
AAA diameter + clinical risk factors + PWS	0.23 (−0.10, 0.56)	0.164
AAA diameter + clinical risk factors + PWRI	0.43 (0.10, 0.76)	0.011

*NRI*, net reclassification index; *CI*, confidence intervals; *PWS*, peak wall stress; *PWRI*, peak wall rupture index. Clinical risk factors included diabetes and current smoking

### Sensitivity analysis in which PWS and PWRI were estimated using a standardized BP

Using PWS and PWRI estimated using a standardized blood pressure of 140/80 mmHg did not substantially change findings from the main analysis (Supplementary Tables 2 to 4).

## Discussion

The main finding of this investigation was that both higher PWS and PWRI were associated with a higher risk of AAA events after adjustment for other risk factors. PWRI was identified as the best risk stratification measure of AAA events in the CART analysis. When compared to AAA diameter alone, PWRI, but not PWS significantly improved the classification of risk of AAA events. Similarly, models including clinical risk factors, AAA diameter, and PWRI also improved the classification of the risk of AAA events compared to diameter alone. The findings are commensurate with a recent meta-analysis of case–control studies that reported that PWRI, but not PWS, was greater in ruptured than asymptomatic intact AAAs of similar diameter [9]. Overall, the findings suggest that PWRI can independently predict AAA events and may add to AAA diameter in stratifying the risk for events in patients with small AAAs. PWRI could potentially assist clinicians in identifying small AAA patients who may benefit from more frequent follow-up or better medical management; however, larger studies are required to investigate this.

Maximum aortic diameter is used in clinical practice to determine when AAA repair should be recommended but has a number of limitations including substantial measurement error [1, 7]. Biomechanical measurements have been proposed for predicting AAA progression but the evidence to support them has been limited [9, 19]. PWS and PWRI are among the most widely studied biomechanical measures [9, 19, 20] although all prior investigations have been of retrospective and case–control design, had small sample sizes, and focused on large AAAs [9, 19]. The current investigation had a number of strengths in comparison to these prior studies such as the inclusion of prospectively collected data and the study of individuals with small AAAs. While the main analysis used patient-specific blood pressure to compute PWS and PWRI, a sensitivity analysis using standardised blood pressure was also performed. It remains unclear which method is most appropriate [9]; nevertheless, the findings were similar in both analyses. Furthermore, a sub-analysis restricted to female participants demonstrated a similar finding to the main analysis.

Although PWS and PWRI can be performed using semi-automated methods [11, 15], FEA is time and resource intensive (~40 min per CTA scan [13, 18]) in comparison to other simpler measures of rupture risk such as AAA diameter [9, 14]. It is therefore important that biomechanical measures have a demonstrated benefit in predicting events to support their use in clinical practice. The current study suggested that PWRI was independently predictive of AAA events and may improve the classification of the risk of events compared to using AAA diameter alone. Further larger observational studies with longer follow-ups are required to confirm or refute the findings of this study.

This investigation has a number of limitations including its small sample size and relatively short follow-up time which was comparable to a recent observational study [10]. The decision to perform surgical repair was at the discretion of the treating vascular surgeon and a standardised protocol was not followed for this study [4]. AAA diameter prior to repair could not be reliably obtained from medical records given the substantial variability in AAA diameter measurement methods and reporting used in routine clinical practise [7]. Importantly there are a number of limitations of FEA, which need to be addressed [9, 19, 20]. Firstly, there remains no standardised approach by which FEA is performed and significant heterogeneity in methods has been reported in prior reviews [9, 19]. Furthermore, there is currently no accurate method by which wall thickness and strength can be estimated from CTA [9, 28]. In the current study, aortic wall strength was estimated from previously reported tensile testing of human wall specimens [11, 14, 16] and a standardised wall thickness was assumed for PWRI estimates. Recent studies suggest that wall thickness can be estimated from MRI [10, 13]; however, this may not be feasible in routine clinical practice. Some factors which may influence aortic biomechanical forces were not investigated in this study such as intra-luminal thrombus and aneurysm flow volume [39, 40]. Lastly, participants were recruited from a limited number of vascular centres, and further investigation is needed to examine whether the findings are repeatable in other populations.

In conclusion, this study suggested that PWS and PWRI can independently predict the risk of AAA events in individuals with small aneurysms. PWRI, but not PWS, significantly improved the stratifying of risk of events compared to aortic diameter alone.

## Supplementary Information

Below is the link to the electronic supplementary material.

Supplementary file1 (DOCX 28 KB)

## Abbreviations

3D: Three-dimensional
AAA: Abdominal aortic aneurysm
BP: Blood pressure
CART: Classification and regression tree analysis
CHD: Coronary heart disease
CI: Confidence intervals
COPD: Chronic obstructive pulmonary disease
CTA: Computed tomography angiography
FEA: Finite element analysis
HR: Hazard ratio
MRI: Magnetic resonance imaging
NRI: Net reclassification index
PWRI: Peak wall rupture index
PWS: Peak wall stress
ROI: Region of interest

## Declarations

### Guarantor

The scientific guarantor of this publication is Jonathan Golledge.

### Statistics and biometry

No complex statistical methods were necessary for this paper.

### Methodology

• prospective

• observational

• multicenter study
