# Supplementary material for: Association between aortic peak wall stress and rupture index with abdominal aortic aneurysm–related events
Source: Eur Radiol. 2023 Mar 10;33(8):5698–706. doi: 10.1007/s00330-023-09488-1 (PMC10326087; doi:10.1007/s00330-023-09488-1)
Supplement: Supplementary file 1 — Supplementary file1 (DOCX 28 KB) [file 330_2023_9488_MOESM1_ESM.docx]

**Association between aortic peak wall stress and rupture index with abdominal aortic aneurysm related events**

**Supplemental Appendix**

**Supplementary table 1.** Association between PWS and PWRI with AAA events in female participants.

**Supplementary table 2.** PWS and PWRI of participants with small AAAs who experienced an AAA event and those who did not, using a standardized blood pressure of 140/80mmHg.

**Supplementary table 3.** Association between PWS and PWRI with AAA events in individuals with small AAA, using a standardized blood pressure of 140/80mmHg.

**Supplementary table 4.** Discrimination and reclassification using PWS and PWRI for AAA events, using a standardized blood pressure of 140/80mmHg.

**Acknowledgements.** Full list of names of TEDY principal investigators and trial coordinators.

**Supplementary table 1.** Association between PWS and PWRI with AAA events in female participants.

|  | **AAA events (AAA rupture or repair)** | | |
| --- | --- | --- | --- |
|  | **Hazard ratio (HR) †** | **95% CI** | **p-value** |
|  | Unadjusted analysis | | |
| PWS (kPa) | 1.57 | 0.75, 3.26 | 0.228 |
| PWRI | 2.24 | 1.07, 4.71 | **0.033** |
|  | Adjusted analysis | | |
| PWS (kPa) | 1.75 | 0.80, 3.84 | 0.162 |
| PWRI | 2.32 | 1.01, 5.32 | **0.047** |

*Adjusted for variables that were found to be different (p<0.100) between participants who had events and those who did not have events (i.e Aspirin and calcium channel blocker prescription); † Hazard ratios expressed per 1 standard deviation increase in PWS or PWRI.

**Supplementary table 2.** PWS and PWRI of participants with small AAAs who experienced an AAA event and those who did not, using a standardized blood pressure of 140/80mmHg.

|  | No AAA event  (n=165) | AAA event  (n=45) | P-value |
| --- | --- | --- | --- |
| PWS (kPa) | 158.9 (143.6, 180.5) | 179.4 (153.3, 202.2) | **<0.001** |
| PWRI | 0.373 (0.311, 0.439) | 0.450 (0.360, 0.566) | **<0.001** |

PWS, peak wall stress; PWRI, peak wall rupture index. Continuous data are presented as median [interquartile range] and were compared using Mann-Whitney U test. P-values highlighted in bold indicate significant differences.

**Supplementary table 3.** Association between PWS and PWRI with AAA events in individuals with small AAA, using a standardized blood pressure of 140/80mmHg.

|  | **AAA events (AAA rupture or repair)** | | |
| --- | --- | --- | --- |
|  | **Hazard ratio (HR) †** | **95% CI** | **p-value** |
|  | Unadjusted analysis | | |
| PWS (kPa) | 1.79 | 1.45, 2.22 | **<0.001** |
| PWRI | 1.95 | 1.55, 2.46 | **<0.001** |
|  | Adjusted analysis | | |
| PWS (kPa) | 1.46 | 1.11, 1.92 | **0.007** |
| PWRI | 1.71 | 1.27, 2.29 | **<0.001** |

*Adjusted for variables that were found to be different (p<0.100) between participants who had events and those who did not have events (i.e AAA diameter, statin prescription, and age); † Hazard ratios expressed per 1 standard deviation increase in PWS or PWRI.

**Supplementary table 4.** Discrimination and reclassification using PWS and PWRI for AAA events, using a standardized blood pressure of 140/80mmHg.

| **Models** | **NRI (95% CI)** | **P-value** |
| --- | --- | --- |
| AAA diameter (reference) | - | - |
| AAA diameter + PWS | 0.18 (-0.15, 0.51) | 0.291 |
| AAA diameter + PWRI | 0.44 (0.11, 0.77) | **0.008** |
| AAA diameter + clinical risk factors + PWS | 0.21 (-0.12, 0.54) | 0.212 |
| AAA diameter + clinical risk factors + PWRI | 0.50 (0.17, 0.83) | **0.003** |

NRI, net reclassification index; CI, confidence intervals. Clinical risk factors included diabetes and current smoking.

**Acknowledgements.** Full list of names of TEDY principal investigators and trial coordinators.

**Australia**

**Gosford Vascular Services, New South Wales**

Principal Investigator Dr Bernie Bourke

Trial Coordinator Dr Michael Bourke

**Queen Elizabeth Hospital, Adelaide, South Australia**

Principal Investigator Professor Robert Fitridge

Trial Coordinators Ms Ruth Battersby and Dr. Prue Cowled

**Royal Brisbane and Women’s Hospital, Queensland**

Principal Investigator Professor Jason Jenkins

Trial Coordinator Dr Sophie Rowbotham and Dr Brad Stefanovic

**The Townsville University Hospital and the Mater Hospital Pilmico, Queensland**

Principal Investigators Professor Jonathan Golledge and Dr Frank Quigley

Trial Coordinators Ms Jenna Pinchbeck and Ms Barbara Bradshaw

**The Netherlands**

Trial Coordinator Ms Stephanie Tomee

**Leiden University Medical Centre, Leiden**

Principal Investigators Associate Professor Jan Lindeman and Prof. Jaap F. Hamming

**Jeroen Bosch Hospital, Hertogenbosch**

Principal Investigator Dr. Jan Willem Hinnen

**Deventer ziekenhuis**

Principal Investigator Dr Robert B van Tongeren

**Haga Ziekenhuis, Den Haag**

Principal Investigator Dr. Jan J Wever

**Haaglanden Medisch Centrum, Den Haag**

Principal Investigator Dr. Daniël Eefting

**Sint Franciscus Gasthuis en Vlietland, Rotterdam**

Principal Investigator Dr. Jerome P van Brussel,

**Antonius Ziekenhuis Nieuwegein**

Principal Investigator Dr. Jean-Paul de Vries (current address: University Medical Center Groningen)

**Elisabeth Tweesteden Ziekenhuis, Tilburg**

Principal Investigator Dr. Patrick W. Vriens

**The United States of America**

**Veterans Administration Hospital and Stanford University, Palo Alto, California**

Principal Investigators Professor Ron Dalman and Dr. Oliver A. Aalami

Trial Coordinator Ms Lori McDonnell
